# Suppression of injury-induced epithelial-mesenchymal transition in a mouse lens epithelium lacking tenascin-C

**Published:** 2010-07-01

**Authors:** Sai-ichi Tanaka, Takayoshi Sumioka, Norihito Fujita, Ai Kitano, Yuka Okada, Osamu Yamanaka, Kathleen C. Flanders, Masayasu Miyajima, Shizuya Saika

**Affiliations:** 1 Department of Ophthalmology, Wakayama Medical University, Wakayama, Japan; 2 Laboratory of Cancer Biology of TGF-β Section, Laboratory of Cancer Biology and Genetics, National Cancer Institute, Bethesda, MD; 3 Animal Laboratory, Wakayama Medical University, Wakayama, Japan

## Abstract

**Purpose:**

To investigate the role of tenascin-C in epithelial-mesenchymal transition (EMT) of the lens epithelium during wound healing in mice. Tenascin-C is a component of the extracellular matrix in patients having post-operative capsular opacification.

**Methods:**

The crystalline lens was injured by needle puncture in tenascin-C null (KO, n=56) and wild-type (WT, n=56) mice in a C57BL/6 background. The animals were killed at day 2, 5, or 10 post-injury. Immunohistochemistry was employed to detect α-smooth muscle actin (αSMA), a marker of EMT, collagen type I, transforming growth factor β1 (TGFβ1), phospho-Smad2, phospho-adducin, and phospho-myosin light chain 9 (MLC9). The expression levels of phospho-adducin and phospho-MLC9 were used as markers for the activation of protein kinase C and Rho kinase, respectively.

**Results:**

The expression of tenascin-C was upregulated in WT lens epithelial cells adjacent to the capsular break at day 5. The results showed that injury-induced EMT of the mouse lens epithelium, as evaluated by histology and the expression patterns of αSMA and fibronectin, was attenuated in the absence of tenascin-C. Upregulation of TGFβ1 expression in the epithelium was also inhibited, and loss of tenascin-C attenuated the phosphorylation of Smad2 and adducin in epithelial cells adjacent to the capsular break. The expression of phospho-adducin was suppressed, while the expression level of phospho-MLC9 was unchanged, in the healing epithelium in the absence of tenascin C.

**Conclusions:**

Tenascin-C is required for injury-induced EMT in the mouse lens epithelium. The mechanism behind this might involve impaired activation of cytoplasmic signaling cascades; i.e., TGFβ/Smad and protein kinase C-adducing signaling, in the absence of tenascin-C.

## Introduction

Upon injury to the anterior capsule, the lens epithelial cells transdifferentiate into fibroblastic cells, which are capable of expressing fibrous extracellular matrix (ECM) components during the healing process. This process is called the epithelial-mesenchymal transition (EMT) [1,2]. However, EMT often leads to the formation of scar tissue (post-operative capsular opacification, PCO), which impairs post-operative vision, rather than the regeneration of normal lens tissue [3-7]. The expression of α-smooth muscle actin (αSMA) and fibrous collagen is characteristic of myofibroblasts [8-10], and these molecules are also established markers of EMT in lens epithelial cells [3,4]. It has been well established that EMT of in vivo lens epithelial cells and other cell types is induced by transforming growth factor β (TGFβ) [2,7,11-18]. Although various signaling cascades are involved in gene expression regulation in EMT, the Smad2/3 and Rho kinase cascades, both of which can be activated by TGFβ, are the two major pathways.

Various ECM molecules; i.e., laminin, collagen types I and III, lumican, osteopontin, fibronectin, etc., are upregulated in lens epithelial cells post-EMT [8-10]. In addition, the TGFβ signaling EMT involved in various cell types is further modulated by ECM molecules expressed by the cells themselves; i.e., fibronectin, lumican, collagen types I and III, fibulin-5, and osteopontin [19-30]. Integrin-derived signaling activated by matrix binding is also required for EMT.

Tenascin-C is a disulfide-bonded hexamer of a matrix component composed of subunits with molecular weights in the range of 120–300 kDa [31,32]. Tenascin-C expression is also upregulated in post-EMT lens epithelial cells in human PCO [33], suggesting a role for tenascin-C in EMT. A similar mechanism has been postulated for the progression/acquisition of invasive characteristics by cancer cells, which is considered to be a type of EMT; tenascin-C is abundantly detected in the invasive margin of cancers, where the cancer cells express an intermediate filament, vimentin, a marker of EMT in neoplastic cells [34,35]. Moreover, it was reported that the absence of tenascin-C suppresses the development of experimental liver fibrosis in mice [36]. The mechanism behind this involved the attenuation of fibroblast-myofibroblast conversion and presumably EMT of hepatocytes associated with reduced expression of fibrogenic cytokines. This finding prompted us to hypothesize that loss of this molecule also attenuates EMT in vivo; i.e., in the lens epithelium post-injury, because both EMT and fibroblast-myofibroblast conversion produce myofibroblasts, the key player in tissue fibrosis formation. To address this question in the present study, we took advantage of the availability of tenascin-C null (KO) mice [37], and showed that injury-induced EMT of the mouse lens epithelium was perturbed in KO mice.

## Methods

Our experiments were approved by the DNA Recombination Experiment Committee and the Animal Care and Use Committee of Wakayama Medical University, Wakayama, Japan and were conducted in accordance with the Association for Research in Vision and Ophthalmology Statement for the Use of Animals in Ophthalmic and Vision Research.

### Lens injury in mouse eyes

Lens injury was performed as previously reported [4,17,31]. We employed the protocol for mouse lens capsular injury approved by National Cancer Institute/National Institutes of Health, Bethesda, MD (Laboratory of Cell Regulation and Carcinogenesis). KO mice with a C57Bl/6 background (n=56) and wild-type (+/+, WT, n=56) mice were generally anesthetized with an intraperitoneal (i.p.) injection of pentobarbital sodium (70 mg/kg bodyweight) and were then topically administered oxybuprocaine eye drops [4,17,26]. Our preliminary investigation showed that the incidence of EMT in the lens epithelium post-injury was similar between the WT and heterozygous mice (data not shown). A small corneal incision was made in the central anterior capsule of one eye (right) with a 26 G hypodermic needle through a corneal incision after topical application of mydriatics, as previously reported [4,17,26]. In brief, the central anterior lens capsule was pierced once with the blade of the 26 gauge needle. The depth of puncture from the corneal surface was approximately 300 μm, which was about one quarter of the length of the needle blade. The animals that accidentally received a deeper lens injury were not included in the experiment. After instillation of ofloxacin ointment, the mice were allowed to heal for 2, 5, or 10 days. The number of eye samples were 20/20, 21/21/, and 21/21 for the WT/KO mice at 2, 5, and 10 days, respectively. Most of the enucleated globes (16/16, 17/17, and 17/17 for the WT/KO mice at 2, 5, and 10 days, respectively) were fixed and embedded in paraffin, and the rest were embedded in optimal cutting temperature (OCT) compound for cryosectioning, as previously reported [4,17,26].

### Histology and immunohistochemistry

Deparaffinized sections and fixed cultured cells were processed for immunohistochemistry as previously reported [4,17,26]. The antibodies used were anti-tenascin C antibody (1:100 in PBS; Chemicon, Temecura, CA), mouse monoclonal anti-αSMA antibody (1:100 in PBS; Neomarker, Fremont, CA), goat polyclonal anti-fibronectin antibody (1:100 in PBS; Southern Biotechnology, Birmingham, AL), goat-polyclonal anti-phospho-Smad2 antibody (1:100 in PBS; Chemicon), goat polyclonal anti-phospho-adducin (Ser 726) antibody (1:200 in PBS; Santa Cruz Biotechnology, Santa Cruz, CA), goat polyclonal anti-TGFβ1 antibody (1:200 in PBS; Santa Cruz Biotechnology), goat polyclonal anti-TGFβ2 antibody (1:200 in PBS; Santa Cruz Biotechnology), goat polyclonal anti-TGFβ receptor type I (TGFβ-RI) antibody (1: 200 in PBS, Santa Cruz Biotechnology), and goat polyclonal anti-TGFβ receptor type II (TGFβ-RII) antibody (1:200 in PBS; Santa Cruz Biotechnology). After being allowed to react with the secondary peroxidase-conjugated antibody and washed in PBS, the antibody complex was visualized using 3,3′-diaminobenzidine. Immunostaining for TGFβ1 was performed as previously reported [17]. Activation of the protein kinase C cascade was assayed by immuno-detection of phospho-adducin (Ser 726). Activation of Rho kinase signaling was evaluated by immuno-detection of phospho (Thr 18/Ser 19)-myosin light chain 9 in cryosections (7 μm in thickness) fixed in cold acetone. After nuclear counterstaining with methyl green, the specimens were observed under regular light microscopy.

## Results

### Immunohistochemistry for tenascin-C

**Figure 1:**
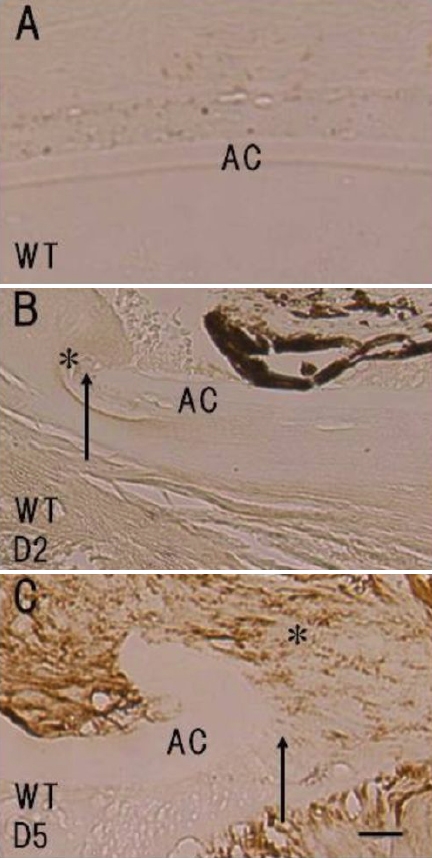
Expression of tenascin-C protein in the injured mouse lens epithelium. Neither the epithelium of an uninjured lens (**A**) nor that of an injured lens at day 2 post-injury (**B**, asterisk) was labeled for tenascin-C. **C**: Immunoreactivity for tenascin C was observed in the lens epithelium (asterisk) adjacent to the break in the anterior capsule at day 5 post-injury. The arrows indicate the edge of the break in the anterior capsule. Bar, 10 μm.

As tenascin-C expression is reportedly upregulated in human lens epithelial cells during PCO, we first examined whether mouse lens epithelial cells express tenascin-C post-injury using immunohistochemistry. No immunoreactivity for tenascin-C was detected in the uninjured lens epithelia or the injured lens epithelia at day 2 post-injury, but tenasin-C immunoreactivity was observed in the lens epithelium cells adjacent to the break in the anterior capsule at day 5 post-injury (Figure 1). The KO lens epithelium did not expressed tenascin-C post –injury (data not shown).

### Histology of injured mouse lenses

**Figure 2:**
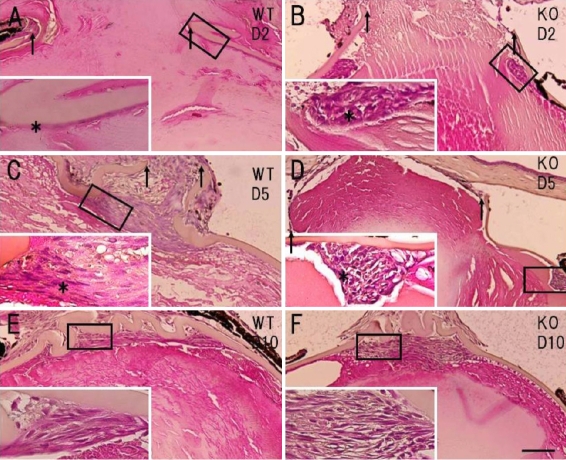
Histology of the edge of the break in the anterior lens capsule in wild-type (WT) and tenascin-C null (KO) mice. At day 2, the lens epithelial cells around the capsular break (arrows) exhibited an epithelial-like morphology in both the WT (**A** and insert; asterisk) and KO (**B** and insert; asterisk) mice. At day 5, the epithelial cells had formed a multilayer at the edge of the broken capsule in both the WT (**C**) and KO (**D**) mice. The wound indicated by the arrows was filled with cells in the WT mice (**C**), while that in the KO mouse lens remained open (**D**). At a higher magnification, the WT cells (**C**, insert) showed an elongated fibroblast-like shape (asterisk), while those of the KO lens (**D**, insert) exhibited an epithelial-like morphology (asterisk). At day 10, a multi-cellular layer had formed beneath the anterior capsule in both the WT (**E**) and KO (**F**) mice. At a higher magnification, the cells in both groups (**E** and **F**; inserts) exhibited an elongated fibroblast-like shape. Inserts of **A** to **F** show the higher magnification images of the boxed areas in **A** through **F**. AC, anterior lens capsule, Bar, 10 μm (**A** through **F**), or 25 μm for each of the inserts.

We examined the effects of endogenous tenascin-C on injury-induced EMT of the lens epithelium using HE staining . The histology of the HE-stained tissues showed a marked alteration in the morphology of the cells at the injury site in the WT mice (Figure 2). The lens epithelial cells of the uninjured lens and those at the capsular break at day 2 post-injury maintained a non-elongated morphology in both the WT (Figure 2A and insert) and KO mice (Figure 2B and insert). At day 5 (Figure 2C and insert) and 10 (Figure 2E and insert), the WT cells exhibited an elongated, fibroblast-like morphology in all the specimens examined, suggesting that EMT had occurred. At these time points, the lens capsule adjacent to the break was folded, presumably by contraction of the myofibroblasts in the WT tissues, while it did not appear to be folded or contracted in the KO tissues. On the other hand, the KO lens epithelial cells were of epithelial shape, even at day 5, in all specimens examined (Figure 2D and insert). Finally, the KO cells exhibited a fibroblastic, elongated morphology at day 10 (Figure 2F and insert).

### Immunohistochemistry for EMT-related components

**Figure 3:**
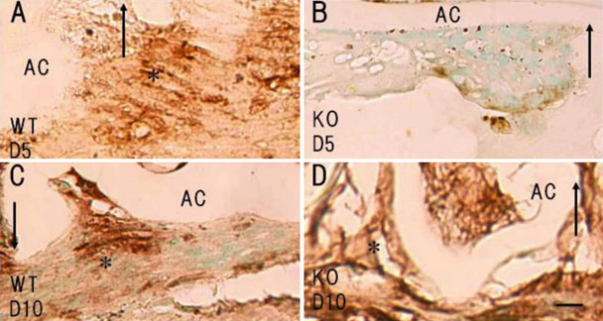
Expression pattern of α-smooth muscle actin (αSMA) in lens epithelial cells of injured lenses. The multicellular layer of epithelial cells at the edge of the broken capsule was strongly labeled with anti-αSMA antibody in a wild-type mouse at days 5 (**A**, asterisk) and 10 (**C**, asterisk). On the other hand, in a tenascin-C null (KO) mouse almost none of the epithelial cells in the multicellular layer were stained for αSMA at day 5 (**B**). At day 10, the majority of the cells were labeled in the KO mouse (**D**, asterisk). Arrows, edges of the injured anterior capsule. AC, anterior capsule; Bar, 10 μm.

HE histology suggested that loss of tenascin-C attenuated the EMT process in the lens epithelium. Therefore, we conducted immunodetection of αSMA, fibronectin, and TGFβ1 to confirm this hypothesis. Our previous papers demonstrated the upregulation of the lens epithelium αSMA protein level at day 5 post-injury. Both the WT and KO epithelial cells of the uninjured lenses and those of the injured lenses from each mouse type were negative for αSMA at day 2 (not shown). The multi-layered fibroblast-like lens cells observed at the capsular break in the WT mice were markedly labeled for αSMA at day 5 (Figure 3A), whereas the epithelial-shaped lens cells in 15 of 17 KO mice were negative for αSMA at this time point (Figure 3B). At day 10, the majority of the cells beneath the broken capsule were labeled with anti-αSMA antibody both in the WT and the KO lenses (Figure 3C,D). As shown by HE histology, at day 5 an area of the lens capsule adjacent to the break was folded, presumably by the contraction of myofibroblasts in the WT tissues, while folding or contraction was seen in the KO tissues.

**Figure 4:**
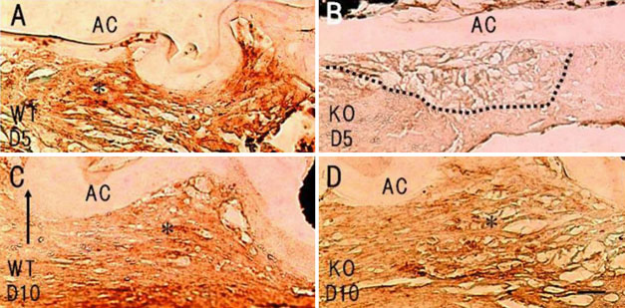
Expression pattern of fibronectin in injured lenses. At days 5 (**A**) and 10 (**C**), immunoreactivity for fibronectin was detected in the multicellular layer formed at the site of capsular injury in a wild-type mouse (**B**), whereas no such immunoreactivity was observed in the multicellular layer at day 5 in an injured tenascin-C null lens, although it was readily detected at day 10 (**D**). Arrows, edge of the injured anterior capsule. AC, anterior capsule; Bar, 10 μm.

Fibronectin is a major adhesive glycoprotein involved in wound healing. Immunostaining clearly showed that fibronectin protein expression in the lens epithelial cells of the injured lens was inhibited in the KO mice. At day 2, no fibronectin was detected in the injured lens epithelia of the WT or KO mice (not shown). At day 5, loss of tenascin-C suppressed the upregulation of fibronectin expression in the injured lens epithelium; lens cells of a fibroblastic morphology (that were labeled for αSMA) were labeled for fibronectin in WT mice (Figure 4A), although the cells in the KO mice were negative (Figure 4B). At day 10, the lens epithelia in the injured lenses of both the WT and KO mice were positive for fibronectin (Figure 4C,D).

**Figure 5:**
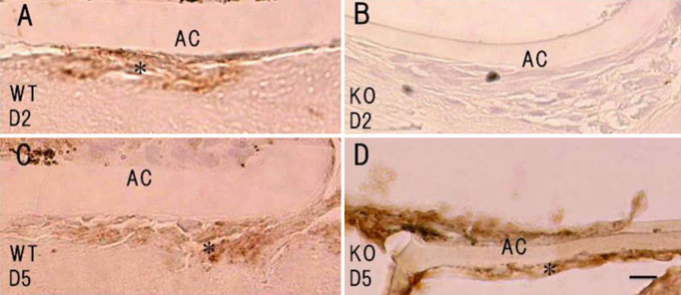
Expression pattern of transforming growth factor β1 (TGFβ1) in injured lenses. At days 2 (**A**) and 5 (**C**), immunoreactivity for TGFβ1 was detected in the lens epithelial cells adjacent to the site of capsular injury in a wild-type mouse. Although no such immunoreactivity was observed in the multicellular layer at day 2 in an injured tenascin-C null lens, it was readily detected at day 10 (**D**). Arrows, edge of the injured anterior capsule. AC, anterior capsule; Bar, 10 μm.

Although we previously reported that the most important type of TGFβ for the activation of Smad signaling in the injured mouse lens epithelium is TGFβ2 derived from the aqueous humor [4], there is a possibility that autocrine TGFβ1 also affects these cells. The present immunohistochemical analysis did not detect TGFβ1 in the uninjured lens epithelium, as previously reported (data not shown) [17]. TGFβ1 expression (Figure 5A) was upregulated in the EMT lens epithelium in the WT mice, while this upregulation was quite minimal in the KO mice at day 2. At day 5, the anterior lens epithelium adjacent to the capsular break was labeled for TGFβ1 in both the WT and KO mice (Figure 5C,D). TGFβ2 was detected in the whole the lens epithelium of the uninjured and healing lenses in both the WT and KO mice (data not shown). TGFβ2 was constitutively its expressed in the lens epithelium, and its expression pattern in the healing epithelium was not affected by the loss of TNC (data not shown).

**Figure 6:**
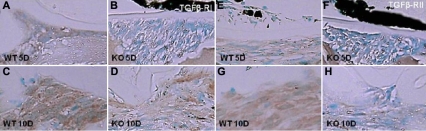
Expression patterns of transforming growth factor β-receptor type I and type II in an injured lens epithelium. At day 5 post-injury, multilayered lens epithelial cells in an injured lens were markedly labeled with anti-TGFβ-RI in a wild type (WT; **A**) mouse, while its immunoreactivity was quite faint in the cells of a tenascin C null (KO; **B**) mouse. At day 10, the cells of the mutilayer beneath the anterior lens capsule were labeled for TGFβ-RI in both the WT and KO mice (**C**, **D**). At day 5 post-injury, the multilayered lens epithelial cells in an injured lens were markedly labeled with anti-TGFβ-RII in a WT mouse (**E**), while its immunoreactivity was quite faint in the cells of a KO mouse (**F**). Even at day 10, immunoreactivity for TGFβ-RII was more prominent in the WT cells than in the KO cells (**G**, **H**); Bar, 10 μm.

Regarding the TGFβ receptors, we examined the expression patterns of TGFβ-RI and TGFβ-RII in the injured lens epithelium using immunohistochemistry. Both the WT and KO lens uninjured lens epithelia were very faintly labeled with antibodies against TGFβ-RI and TGFβ-RII (data not shown). At day 5 post-injury, multilayered lens epithelial cells in the injured lenses were markedly labeled with anti-TGFβ-RI in the WT mice, while its immunoreactivity was quite faint in the cells from the KO mice (Figure 6A,B). At day 10, the cells in the mutilayer beneath the anterior lens capsule were labeled for TGFβ-RI in both the WT and KO mice (Figure 6C,D). At day 5 post-injury, the multilayered lens epithelial cells in the injured lenses were markedly labeled with anti-TGFβ-RII in the WT mice, while its immunoreactivity was quite faint in the cells from the KO mice (Figure 6E,F). Even at day 10, the immunoreactivity for TGFβ-RII was more prominent in the WT cells than in the KO cells (Figure 6G,H). TGFβ-related signaling mediators. As for the TGFβ-related signaling cascades, we examined whether Smad2 and adducin were phosphorylated in the epithelia examined. Adducin is a membrane cytoskeletal protein that is phosphorylated in association with the activation of multiple signaling pathways such as the Rho kinase and protein kinase C pathways [38].

**Figure 7:**
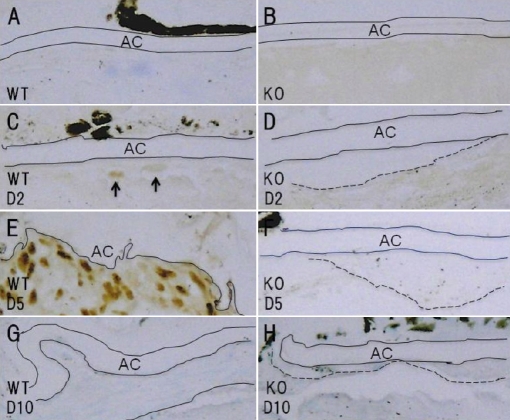
Expression patterns of phospho-Smad2 in the lens epithelia of injured lenses. Uninjured lens epithelial cells beneath the anterior capsule were not labeled for phosph-Smad2 in either the wild-type (WT; **A**) or tenascin-C null (KO; **B**) mice. At day 2, the nuclei of some epithelial cells were labeled for phospho-Smad2 in an injured WT lens (**C**, arrows). On the other hand, in a KO injured lens the nuclei of the epithelial cells at the leading edge of the cellular multi-layer were not labeled for phospho-Smad2 (**D**). At day 5, many epithelial cells were founded to contain nuclei that were positive for phospho-Smad2 in the multicellular layer at the capsular break site in a WT mouse (**E**, arrows), whereas no phospho-Smad2 was detected in the majority of epithelial cells in multicellular layer in an injured KO lens (**F**). At day 10, the cells around the break in the anterior capsule were not labeled for phospho-Smad2 in either the WT (**G**) or KO (**H**) mice. Dotted lines, border between the multilayered cells and the lens cortex, AC, anterior capsule; Bar, 20 μm.

The uninjured lens epithelia of the WT and KO mice were negative for phospho-Smad2 (Figure 7A,B). At day 2, lens epithelial cells containing nuclei labeled for phospho-Smad2 were observed adjacent to the break in the anterior capsule in the WT mice (Figure 7C), whereas the epithelial cells in the KO mice were not labeled (Figure 7D). At day 5, many cells with phospho-Smad2-positive nuclei were observed around the capsular break in the WT mice (Figure 7E). At this time point, the majority of the lens epithelial cells in the cell multi-layer that had formed adjacent to the break in the KO mice were negative for phospho-Smad2 (Figure 7F). At day 10, the cells around the break in the anterior capsule were not labeled for phospho-Smad2 in either the WT or KO mice (Figure 7G,H).

**Figure 8:**
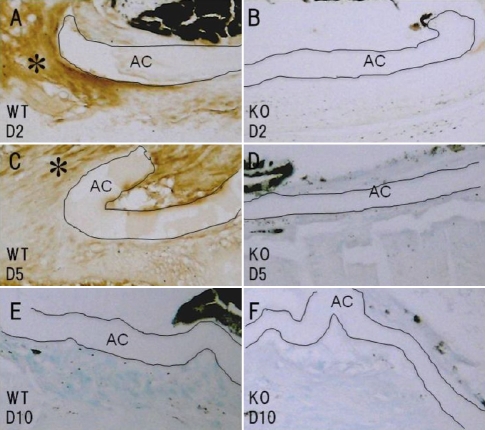
Expression patterns of phospho-adducin in the lens epithelia of injured lenses. At day 2, many lens epithelial cells were labeled for phospho-adducin in an injured wild-type (WT) lens (**A**, asterisk). On the other hand, in a tenascin-C null (KO) injured lens the epithelial cells at the leading edge were not labeled for phospho-adducin (**B**). At day 5, many epithelial cells were positive for phospho-adducin in the multicellular layer at the capsular break site in a WT mouse (**C**, asterisk), although its immunoreactivity was weaker than that in the cells at day 2, whereas phospho-adducin was only faintly detected in the majority of epithelial cells (star) in an injured KO lens at day 5 (**D**). At day 10, the cells around the break in the anterior capsule were not labeled for phospho-adducin in either WT (**E**) or KO (**F**) mice; Bar, 10 μm.

We next immunostained phospho-adducin.38 The uninjured lens epithelia of the WT and KO mice were negative for phospho-adducin (data not shown). At day 2, lens epithelial cells labeled for phospho-adducin were seen in the cytoplasm adjacent to the break in the anterior capsule in the WT mice (Figure 8A), whereas no epithelial cells were labeled for phosoho-adducin in the KO mice (Figure 8B). At day 5, the majority of the lens epithelial cells in the cell multi-layer that had formed adjacent to the break in the KO mice were still negative for phospho-adducin (Figure 8D). At this time point, many elongated cells contining phospho-adducin-positive cytoplasm were observed around the capsular break in the WT mice (Figure 8C). At day 10, the cells around the break in the anterior capsule were not labeled for phospho-adducin in either the WT or KO mice (Figure 8G,H).

**Figure 9:**
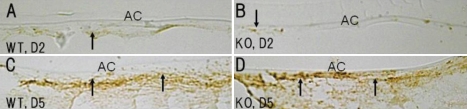
Expression patterns of phospho (Thr 18/Ser 19)-myosin light chain 9 in lens epithelia of injured lenses. At day 2, many lens epithelial cells were faintly labeled for phospho-myosin light chain 9 in both an injured wild-type (WT) and a tenascin-C null (KO) injured lens epithelium (**A**, **B**, arrows). At day 5, both the WT and KO cells were obviously immunolabeled for phospho-myosin light chain 9 (**C**, **D**, arrows). AC, anterior epithelium; Bar, 10 μm.

We then evaluated the activation of Rho kinase signaling by immuno-detection of phospho (Thr 18/Ser 19)-myosin light chain 9. Phosphorylated-myosin light chain 9 was faintly detected as early as day 2 in the lens epithelial cells of the injured lenses of both the WT and KO mice and was markedly observed thereafter in these cells in both genotypes (Figure 9). The levels of immunoreactivity in the lens epithelial cells were similar in both genotypes.

## Discussion

The present study was undertaken to elucidate whether tenascin-C plays a significant role in injury-induced EMT of the lens epithelium using tenascin-C-deficient mice. Here, we show that the absence of tenascin-C perturbs injury-induced EMT in mice in vivo. The present study clearly answered this question because the myofibroblasts in healing lens must be derived from the cells inside the lens, e. g., the lens epithelium, without contamination of fibroblastic cells from outside the lens. Since HE histology strongly suggested the impairment of injury-induced EMT in the lens epithelium, we conducted immunohistochemistry and detected reduced expression of αSMA (relative to that in the WT lens), a myofibroblast marker, in lens cells in the KO mice at day 5 post-injury. A similar phenomenon was reported previously; i.e., that tenascin-C is specifically expressed at the invasive fronts of cancers, where neoplastic cells acquire a migratory characteristic during EMT [34,35].

The exact mechanism behind this phenomenon remains to be investigated. We previously reported that TGFβ/Smad signaling is essential for the occurrence of EMT in injured mouse lenses [4,17,26]. The present immunohistochemical analysis showed that the activation of the Smad signal was severely suppressed in the epithelial cells of injured KO mouse lenses compared with that in the epithelial cells of injured WT mouse lenses. TGFβ2, but not TGFβ1, predominates in the aqueous humor [39-41]. We also previously reported that TGFβ1 expression is upregulated in the injured lens epithelium in association with the progression of EMT. On the other hand, blocking TGFβ2, but not TGFβ1, in the aqueous humor abrogates Smad signaling in the the injured mouse lens epithelium [17], suggesting that the upregulation of TGFβ1 expression in the epithlia upon injury might not play a significant role in the activation of Smad signaling. Thus, in the present study, we examined the expression of TGFβ1 in the injured lens epithelium as a marker of the progression of EMT and showed that the upregulation of TGFβ1 expression in the epithelial cells of the injured lens was also attenuated in the absence of tenascin-C. The expression of TGFβ2, as detected by immunohistochemistry, in the lens epithelium was not affected by the absence TNC (data not shown).

Multiple signaling cascades; i.e., the protein kinase C and Rho kinase cascades, are reportedly required for the EMT and the expression of fibrogenic components, but TGFβ and Smad signaling are considered to be essential. Adducin is a membrane-associated actin-binding protein that is phophorylated by protein kinase C at its COOH-terminal region. Protein kinase C is also involved in cell migration by modulating the morphology of the F-actin cytoskeleton [38,42]. We therefore examined whether the pattern of the COOH-terminal (Ser 726) phophorylation of adducin was altered by the loss of tenascin C in the injured lens epithelium. Our results showed that the phosphorylation of adducin in the injured lens epithelium was greatly reduced in the KO mice compared with the WT mice, suggesting that the downregulation of protein kinase C activity in the absence of tenascin C contributes to the attenuation of lens cell EMT in addition to the attenuation of Smad signaling. Rho kinase is another signal that is reportedly essential for EMT in many cell types including the lens epithelium [43,44]. We therefore evaluated the activation of Rho kinase signaling via immuno-detection of phospho (Thr 18/Ser 19)-myosin light chain 9. Phosphorylated-myosin light chain 9 was faintly detected as early as day 2 in the lens epithelial cells of the injured lenses of both the WT and KO mice and was markedly observed thereafter in these cells, suggesting that the absence of tenascin C does not affect Rho kinase activity.

We also examined the expression of TGFβ receptors in the injured lens epithelium to understand the susceptibility of the cells to ligands. Immunohistochemistry showed that lens epithelial cells upregulated the expression of TGFβ-RI and RII as early as day 5 during the healing process and the progression of EMT in the WT mice. On the other hand, as for the expression pattern of αSMA, the upregulation of TGFβ receptor expression was delayed, negative at day 5, and then positive at day 10 in the KO mice. Nevertheless, it is still unclear whether the delayed EMT seen in the KO mice can be ascribed to the suppression of the upregulation of TGFβ receptor expression in the absence of tenascin-C. There is a possibility that delayed upregulation of TGFβ receptor expression in KO mice is responsible for the impairment of the phosphorylation of Smad2 and adducin in KO cells. On the other hand, another possible mechanism of the impairment of these signaling transmitters is that the impairment of integrin-derived signals caused by the loss of tenascin-C affects TGFβ-derived signaling via signaling crosstalk.

It was reported that in the stroma of the eye cornea, injury-induced expression of fibronectin, matrix component essential for tissue repair, is suppressed in the absence of tenascin-C [45]. In addition, integrin-mediated signaling is reportedly required for fibroblast-myofibroblast conversion and EMT in tissue fibrosis. In the present study, reduced expression of fibronectin was also observed in the injured lens epithelia of KO mice, suggesting that impairment of fibronectin-integrin signaling is also involved in the suppression of lens cell EMT in KO mice.

Exploring the mechanism of EMT in the lens epithelium post-injury is necessary to aid our understanding of the pathogenesis of capsular opacification post-cataract surgery. We reported that anti-Smad molecules inhibit EMT in the lens epithelium and suggested their clinical efficacy in the prevention of PCO [46,47]. However, the present study together with previous reports show the important roles of the ECM in the pathogenesis of PCO. Further study is needed to elucidate the matrix component signaling systems that are required for the occurrence of lens cell EMT.
